# An Overview of Clinical Studies on Fiber Post Systems

**DOI:** 10.1155/2013/171380

**Published:** 2013-10-23

**Authors:** Idil Dikbas, Jale Tanalp

**Affiliations:** ^1^Department of Prosthodontics, Faculty of Dentistry, Yeditepe University, Bagdat Caddesi No. 238, Goztepe, Istanbul, Turkey; ^2^Department of Endodontics, Faculty of Dentistry, Yeditepe University, Bagdat Caddesi No. 238, Goztepe, Istanbul, Turkey

## Abstract

Intraradicular posts are useful adjuncts in the restoration of endodontically treated teeth. These systems have undergone a significant evolution in recent years, and fiber-reinforced systems have started to be incorporated into routine clinical care more frequently. Despite the high number of laboratory studies pertaining to the characteristics of fiber posts, clinical studies evaluating their general success rates are rather limited. Since clinical investigations are reliable means to achieve information about the general behavior pattern of materials or techniques, assessment of this data will be beneficial to have a better understanding of fiber-reinforced intraradicular post systems. The purpose of this paper was to make a summary of clinical studies regarding various fiber posts. A PubMed search was conducted and articles dating back to 1990 were retrieved. The paper provides an overview of clinical studies on fiber posts specifically in the last decade as well as commentary analysis.

## 1. Introduction

It is generally the common opinion of dental authorities that endodontically treated teeth are more prone to fracture due to a variety of factors such as extensive tissue loss, loss of moisture content, and flexibility as well as decrease in resistance due to endodontic access preparations [1–3]. Consequently, it is rather common for endodontically treated teeth to receive full coverage restorations to ensure that they show better resistance to external forces [4]. Furthermore, even the rate of complications of prosthetic restorations fixed on endodontically treated abutment teeth has been reported with a higher incidence, resulting in tooth loss [5]. This shows that meticulous care is to be exercised when confronted with challenging cases where endodontically treated teeth are accompanied by hard tissue loss with extensive magnitude.

Usage of intracanal posts is a commonly practiced procedure specifically for challenging cases indicated above. This application is especially preferred for the restoration of endodontically treated teeth where extensive loss of hard tissue necessitates additional reinforcement of the remaining structure apart from the crown itself. 

The most common type of posts used in dentistry has been cast post and cores in the last decade which generally includes an additional laboratory stage where a custom post is prepared according to the impression taken from the prepared post space. In case the practitioner prefers a faster application without an impression-taking stage, prefabricated metallic posts have also been launched to the market, with a wide range of structural designs, serving the requirements of different clinical cases. Screw posts are also available; however, it has been indicated that these should be inserted with caution as they might result in undesirable complications such as vertical root fractures [6].

Although cast or prefabricated metallic posts have been widely used for a long period, some disadvantages and drawbacks have also been determined associated with these systems among which loss of retention, root fractures, corrosion, necessity of removal of extensive root structure, and stress concentration can be given as examples [7]. These drawbacks have driven manufacturers and dental producers to seek for new alternatives, which led to the introduction of carbon, glass, polyethylene, and quartz fiber post systems. One of the most significant features of these systems has been their lower elasticity modulus leading them to behave similarly to dentine and show similar stress patterns under external impacts [8–10].

Consequently, the complications related to these systems have been observed as less catastrophic, which generally includes relatively more reparable problems such as debonding. Additionally, the less invasive and more conservative space preparation required by these systems has been a significant advantage [11].

Although prefabricated fiber posts have been assessed in terms of their mechanical and physical properties through various *in vitro* settings, it is an undeniable fact that the most reliable information regarding their general characteristics can be achieved by clinical studies. No matter how far it is attempted to simulate clinical circumstances,* in vitro* experimental designs are unable to draw one-to-one resemblance to real *in vivo* conditions. Therefore, an analysis of accumulated data by clinical trials is the most feasible means to collect evidence-based information on these systems which are suggested to be more beneficial over metallic-intraradicular structures.

This review article attempted to collect and summarize information accumulated so far pertaining to fiber post systems used in clinical circumstances and critically analyze fiber posts through data gathered by long-term clinical examination. A PubMED search was conducted by inserting keywords “fiber post,” “clinical study,” and “dentistry.” Twenty-four clinical articles were retrieved. Prospective and retrospective long-term clinical studies were among the inclusion criteria whereas review articles, case reports, or studies including the followup of a limited number of cases were excluded based on the assumption that data accumulated through long-term clinical studies are essential for a general practitioner when making clinical decisions [12]. During the review process, the relevant literature was further obtained through the reference sections of the retrieved articles to provide more supportive information. Overall, 32 articles were included.  Table 1 generally summarizes clinical studies included in the review. 

## 2. Chronological Overview of Clinical Studies on Fiber Posts

The history of nonmetallic posts dates back to 1990 with the introduction of Composipost, based on the carbon fiber reinforcement principle [13]. The post system has equally stretched and aligned carbon fibers, solidly attached to a special matrix of epoxy resin. The fibers represent 64% of the structural volume and the matrix, which binds the fibers together, is an epoxy resin [14].

Although the system was launched in 1990, it was not until 1998 that a long-term study was undertaken to assess its clinical performance. Fredriksson et al. [7] evaluated 236 teeth incorporating carbon fiber posts treated during a 1-year period by seven Swedish dental practitioners with a mean restoration time ranging between 27 and 41 months. The assessment generally yielded favorable and promising results with no unsalvageable complications. The extraction rate of the evaluated teeth was only 2% which were unrelated with the post system itself.

Another study evaluating Composiposts was by Glazer [14] who reported the results of a prospective study initiated in 1995 and in which 59 carbon fiber Composiposts cemented with Metabond and built up with Core Paste cores were placed into the teeth of 47 patients. The follow-up period ranged between 6.7 and 45.4 months. A standardized protocol was followed during the preparation of the post spaces as well as the cementation process. There were no fractures. The overall failure rate was 7.7% and the cumulative survival rate was 89.6% at the end of the follow-up period. An interesting result obtained was the higher risk carried by lower premolars in terms of failure. There were only 4 failures reported, and of these, 2 were biologic (periapical pathology) that cannot be directly related to the post system per se and 2 were mechanical which were limited to core and crown debonding and could not be regarded as catastrophic. However, the authors criticized their findings by indicating that the length of the followup was relatively short to make a definite generalization. The authors attempted to explain the greater biologic failure rate among premolars compared with anterior teeth with the more complex root canal system associated with this group of teeth. 

Ferrari et al. [15] also evaluated Composipost from a clinical perspective on 200 patients who were divided into 2 groups receiving either Composiposts or cast post and cores. Composipost system was found to be superior to conventional cast post and core system after 4 years of clinical service with a success rate of 95%. As in other studies, no catastrophic failure was detected and the 5% failure rate was associated with noncompliance of the patient or endodontic failure that was not directly related to the post. On the other hand, 9% of the cases in the conventional cast post and core group revealed irreparable failures such as root fractures. In another study, Ferrari et al. [16] evaluated Composipost again; however, this time by comparing the system with other fiber post systems, Aestheti posts and Aestheti Plus Posts during clinical service ranging from 1 to 6 years. They determined no significant differences between the groups and advocated the routine usage of fiber posts in combination with bonding/luting materials.

Composipost has drawn attention not only as the survival period of teeth where it is used but by also the influence of the overlying restoration type on its survival rate. An example to such an assessment was the study by Mannocci et al. [4] where the clinical success rate of endodontically treated premolars restored with fiber posts and direct composite restorations was compared to those which underwent similar treatment but were restored with full coverage with metal-ceramic crowns for a period of 3 years. No difference was observed in the failure frequencies of the 2 groups nor was there any difference detected between the number of failures caused by tooth decementation and the presence of marginal gaps. The lack of possibility of matching 2 pairs of teeth in the same patient which necessitated the inclusion of one tooth per patient was regarded as a possible drawback of the study that posed some sort of bias. It was promising that no serious type of failure occurred, and the type of complications was limited to reparable ones such as post decementations and marginal gaps revealed by radiographs. The authors also proposed the reason for decementation as a result of water contact due to marginal leakage. Another note made by the authors was the planning of the study until the 6th year. They also drew attention to the fact that in case no serious and catastrophic complication occurred within this period, fiber posts in combination with composites could be advocated as an economic and time saving alternative to the more invasive and expensive full coverage. The evaluation of only premolars was considered as another possible limitation this study posed as the relatively better predictability of direct composite restorations on premolars compared to molars. Nevertheless, the study held significance as it demonstrated the success of fiber direct composite restorations in premolars. In case this can be supported by future research, direct composites reinforced by fiber posts might serve as feasible alternatives for premolars with severe hard tissue loss.

As years progressed, attempts were made to reinforce the existing carbon fiber post systems. CFRC, a reinforced version of carbon fiber, has also been studied clinically and compared with conventional cast posts. The uniqueness of this post system was the alignment of the principal fibers at an angle to the principle axis of the composite, a high elastic modulus and possibility of transverse strength. King et al. [17] conducted a restricted clinical trial to improve and further validate the findings of a previous study where CFRC was assessed under *in vitro* conditions and yielded favorable results in terms of fracture resistance specifically when used in conjunction with a gold core [18]. They restored 27 single-rooted maxillary anterior teeth in 18 patients either with CFRC post or conventional posts and performed followups until 24, 29, 56, and 87 months. Interestingly, the failure rates of the CFRC posts were found to be higher compared to the control group. The authors drew attention to an issue related to carbon posts that were of clinical significance by stating that the strength of carbon posts decreased by as much as one-third when soaked in water for 24 hours prior to testing. They further commented that it is likely that the posts might have absorbed water from the surrounding tissues during clinical function resulting in early failure. They added that interpretation of the results should be made with caution due to the small sample size of the study.

Hedlund et al. [19] compared the clinical performance of Composipost and Endopost which is another carbon fiber post with different morphological characteristics, specifically designed for narrow root canals. They evaluated 48 posts (either type) on 68 patients over an average period of 2.1 years. A failure rate of 3% was determined related to a premolar that was part of a cantilever fixed partial denture and an upper canine. Both failures were reparable and salvaged through recementation where the restorations were functional for another 10 months.

Clinical efficacy of esthetic fiber posts other than carbon fiber started to be clinically evaluated in the year 2003. Monticelli et al. [20] evaluated the clinical performance of 3 types of esthetic posts applied on 225 patients: Aesthetic Plus, DT, and FRC Postec. Different types of luting cements were used in each of the groups. The patients were followed after 6, 12, and 24 months. No significant difference was noted among the 3 systems tested and all were reported as having reliable clinical performance.

 The authors criticized previous retrospective study designs indicating that the variables used in a prospective study design are controlled at the stage of the case selection and experimental groups can be made homogeneous in all but the variable under study. They also stated that such a study model allows the limitation of confounding factors and delivers more reliable and valuable information. Thus, the variables under study, that is, the different materials used for the restoration, became the factors most crucially responsible for the variability in the clinical performance of the teeth over time. This comment of the authors deserves merit as the types of teeth, cementation techniques, and the operator performing the procedures were also standardized, thus allowing only the materials tested to be evaluated from a clinical perspective.

In line with the results of the previous investigations, failure modes were not catastrophic and they were rather salvageable. The authors also reported that as no difference was noted among the 3 translucent post systems tested, the selection of the adhesive-cement combination basically becomes a matter of personal preference of the clinician, based on experience and habits. 

In a prospective study evaluating the clinical performance and acceptability of quartz fiber-reinforced epoxy posts used in endodontically treated teeth over a 30-month period, 180 endodontically treated teeth belonging to 132 patients were restored using Aestheti-Plus quartz-fiber posts. The percentage of failures was reported as only 1.7 over a 30-month period and replacement was possible in all failed cases. In general, these posts systems yielded favorable clinical results with a success rate of 98.3% [21]. An interesting note made by the authors was that a 2 mm ferrule that is an important component for a successful restoration was lacking in cases with adhesive failure. 

Naumann et al. [9] conducted a prospective study and evaluated glass fiber reinforced composite post restorations. The authors performed the study from a different perspective and focused on the shapes of the placed posts, tapered,or parallel sided. One-hundred and five posts received by 83 patients were followed up to a period of 2 years. One and 2 year failure rates of fiber reinforced composite post restorations were 4 and 12%, respectively, and no difference was observed in relative failure frequencies between two different post types. One important feature of this study design was the clear definition of inclusion criteria of patients prior to the study, both in terms of the length of the remaining apical root canal seal and the degree of tooth mobility. Post fractures and loss of post retention were the most frequent failure types, the majority of which were restorable. Naumann et al. [22] in another prospective study assessed two tapered and one parallel-sided posts for the purpose of detecting the major risk factors for failure. In terms of tooth location, higher failure rates were detected in anterior teeth compared to posterior. When the type of tooth contact was taken into consideration, it was determined that teeth with no proximal contacts were more prone to failure compared to those having at least one contact. Also, teeth restored with single crowns were associated with higher failure rates compared to fixed bridges. The authors commented that such a result in terms of the presence of contacts was expected as neighboring teeth helped the distribution of occlusal forces. As for single crowns being more prone to failure, explanation was made as the forces acting on these teeth being in the vestibular-oral direction in spite of the presence of contacts. The failure rates of combined fixed/removable dentures were intermediate compared with single crowns or bridges. The authors commented on such a result as the usage of precision attachments in these systems which might provide additional support as well as the composition of each abutment of at least 2 teeth connected through a fixed bridge. The authors also noted that though attempts were made to incorporate a 2 mm ferrule whenever made possible based on the general consensus that this preparation type may dramatically increase the resistance of post systems, ferrule preparation was not standardized by crown-lengthening procedures, which might pose a limitation in terms of standardization. Crown lengthening is necessary in these cases to ensure a ferrule in case this factor is to be standardized that was one of the missing areas of the investigation. The relatively low number of cases was indicated as another factor that might pose a limitation to make general statements.

Fiber posts (DT Light) were assessed in terms of clinical performance when used with direct resin composites and this combination was proposed as a treatment option that conserves remaining tooth structure in the short term and results in good patient compliance [23].

Schmitter et al. [8] criticized previously performed clinical investigations in terms of lack of standardization and drew attention to the standardization of baseline findings in evaluating the survival rate of a post system. They also commented that the only study available that fulfills the baseline criteria was the one by Naumann et al. [22]. The authors indicated that, in teeth restored with fiber-reinforced posts versus teeth restored with metal screw posts, clinical baseline characteristics besides the post system may influence post survival. They evaluated 100 patients requiring a post for 1-year period and established inclusion criteria and recorded baseline values. Their results showed that fiber reinforced posts had a higher survival rate compared to metallic ones and metallic posts were associated with more unfavorable complications such as root fracture. While the type of tooth and the degree of coronal restoration were factors that impacted on the survival of metallic posts, these parameters had no influence on fiber posts. The authors further commented that because of inhomogeneous study populations and inclusion criteria, and other factors, direct comparison of different studies is difficult. 

In another study evaluating the 2-year outcome of restorative procedures involving the placement of fiber posts in endodontically treated teeth concluded that the major failure types associated with this treatment type were post debonding reported as 4.3% and endodontic failures reported as 3.0% [24]. An important issue drew attention to the fact that even though the restoration seems to be clinically in service, debonding of the adhesive from the resin-infiltrated area and debonding of the resin cement were possible failure types.

Naumann et al. [10] conducted a randomized controlled clinical pilot trial where prefabricated titanium posts were compared with fiber reinforced posts. A self-adhesive resin was used for luting procedures. The study had a detailed and organized study design where baseline criteria such as remaining cavity walls, minimum apical root canal seal, and degree of tooth mobility were well-established. The authors also recorded variable factors such as degree of attrition, number of proximal contacts, antagonistic contacts, and post length within the root canal. Among 87 posts followed, no failures were observed during follow-up period up to 3 years. Though the study concluded that the post material had no influence on success rate, they added that the results should be interpreted with caution as this is a study of short duration and different results may be obtained in longer term trials.

In later years, it is observed that more focus is given on prospective studies with established baseline criteria. A long-term prospective study on 69 patients evaluated Polyethylene fiber-reinforced posts and cores used in endodontically treated teeth over a 97-month period after which high survival rates (95%) were obtained. Moreover, tooth location or type of restorative material had no impact on the overall survival rates [25].

In a short-term study in which cast post and core, carbon fiber reinforced post, and glass fiber reinforced post with composite core restorations were analyzed for a period of 12 months, fiber reinforced post with composite core when used in single rooted upper anterior teeth were found to be associated with a higher success rate in restoration of endodontically treated teeth [26].

Mehta and Millar [27] focused on an issue that is not primarily assessed in clinical studies on fiber post studies the type of cementation. The authors concluded that the choice of cement appears to have a significant role in improving the prognosis based on their results regarding the high failure rate observed (35.9%) where Calibra was used for the placement of restorations.

Signore et al. [28] commented that most existing longitudinal studies of endodontically treated anterior teeth restored with glass-fiber posts in combination with full ceramic crowns were on small series and with followup of limited duration. Therefore, they conducted a longitudinal retrospective study up to 8 years assessing the survival rates of glass fiber posts with parallel-sided or tapered shape. Each type of posts had a high survival rate of 98.48% for an extended period of 5.30 years. Though no difference was found between the shapes of posts in terms of survival rate, the amount of coronal destruction was identified as a variable that had an impact on survival rate, with a higher longevity detected for teeth with 4 or 3 coronal walls.

Another retrospective study was by Ghavamnasiri et al. [29] who evaluated the success rate in endodontic-treated premolars restored with composite resin and fiber reinforced composite posts with ages ranging between 1 and 6 years. Thirty-eight patients with endodontically treated premolar and anterior teeth that were then restored with a coronoradicular quartz fiber post and extensive composite resin restorations were selected for participation in the study. The overall cumulative survival rate (48.8%) was determined, while the survival probabilities after 1, 2, 4, 5, and 6 years of service were 88.37%, 60.95%, 45.71%, 32.65%, and 0%, respectively. The authors also concluded that the dental arch had a significant impact on the survival probability of endodontically treated teeth restored with a quartz fiber post and composite restorations and restorations in maxillary arch were more prone to fail than restorations in mandibular teeth.

When using self-adhesive luted prefabricated posts in severely destroyed abutment teeth with 2 or less cavity walls and a 2-mm ferrule, postendodontic restorations achieved high long-term survival rates irrespective of the post material used (i.e., glass fiber versus titanium).

Glass fiber and metal screw posts were prospectively analyzed over a period of 5 years after which the survival rate of glass fibers was found to be 71.8%. The degree of coronal tooth structure and the post system used posed important factors in terms of risks. On the other hand, some negative statements were also brought in terms of glass fibers over an extended period [6]. Another study incorporating glass fiber posts was by Zicari et al. [30] where these systems were compared with custom-made glass fiber posts or composite cores without posts. Both cast gold and composite fiber post/core systems were found to perform well clinically in the short run.

A long-term study that evaluated 10-year survival of glass-fiber supported prosthodontic restorations revealed relatively high annual failure rate of glass reinforced fiber posts [31]. On the other hand, anterior teeth were more prone to failure and the number of remaining cavity walls was also a critical factor that should be considered whilst placing post systems. 

A recent pilot study [32] compared glass fiber-reinforced epoxy resin posts to titanium posts. 84-month observation revealed comparable survival rates and it was concluded that it was rather the number of cavity walls and the presence of ferrule that were the key factors governing the longevity of the postendodontic restoration, rather than the material used for the post.

## 3. Critical Analysis and Final Comments

This review article attempted to provide an overview of publications in the last decade regarding fiber reinforced posts that draw the attention of a growing number of practitioners recently. Multiple causes of failure need to be analyzed when examining the failure patterns associated with intraradicular post systems such as secondary caries, loss of retention and debonding of the post and crown, root fractures, and distortion of posts as well as post fractures. On the other hand, as observed from this review, clinical investigations performed so far are difficult to compare due to the inconsistencies between sample selections, and established baseline criteria are necessary such as assessment of endodontic treatment, degree of coronal tissue loss, and presence of parafunctional habits. Furthermore; incorporation of a ferrule is very difficult to standardize due to the discrepancies between the types of hard tissue loss associated with each individual case. From this perspective, suggestions can be brought regarding the necessity of standardizing study designs for future trials to obtain more reliable data. Nevertheless, some general statements can be made based on the accumulated data so far such as the favorable and high survival rates associated with fiber post systems. It is also noteworthy to mention that, despite the high number of *in vitro* studies carried out regarding posts, results are difficult to be directly extrapolated to clinical circumstances; therefore, clinical investigations still serve as the best means to have a realistic picture of the behavior of different systems under clinical settings. Long-term prospective studies with well-established baseline criteria will be helpful to further support the already existing data regarding fiber post systems that appear to be favorable alternatives to metallic or ceramic posts specifically due to the salvageable failure characteristics associated with these systems apart from esthetic advantages. 

## Figures and Tables

**Table 1:** The table summarizes general information on clinical studies performed on fiber posts between 1998 and 2012. The term “success” stands for those teeth without any indication of extraction as well as complications such as debonding, post fracture during the conclusion of the serving period.

	Year	Author(s)	Study design	Type of posts assessed	Follow-up period	Success rate (percentage)
1	1998	Fredriksson et al. [7]	Retrospective	Composipost	2-3 years	98

2	2000	Glazer [14]	Prospective	Composipost	6.7–45.4 months	89.6

3	2000	Ferrari et al. [15]	Prospective	Composipost, conventional cast post-core	4 years	95

4	2000	Ferrari et al. [16]	Prospective	Composipost, Aestheti posts, Aestheti-Plus posts	1–6 years	96.8

5	2002	Mannocci et al. [4]	Prospective	Composipost + full cast coverage Composipost + direct composite restorations	1, 2, and 3 years	Composiposts + full cast coverage: 1 year—100 2 year—94.7 3 year—100 Composiposts + direct composite restorations: 1 year—100 2 year—93.8 3 year—98

6	2003	King et al. [17]	Prospective	Carbon fiber post, metallic prefabricated posts	24, 29, 56, and 87 months	Carbon fiber post: 71 Metallic prefabricated posts: 89

7	2003	Hedlund et al. [19]	Retrospective	Composipost, Endopost	2, 3 years	97

8	2003	Monticelli et al. [20]	Prospective	Aestheti Plus (quartz fiber) posts, DT Light posts and FRC Postec posts	2 years	93.8

9	2003	Malferrari et al. [21]	Prospective	Aestheti-Plus posts (quartz fiber)	30 months	98.3

10	2005	Naumann et al. [9]	Prospective	FiberKor posts (3 sizes) (parallel sided and tapered glass fiber)	12 months and 24 months	12 months: 96.2 24 months: 88.6

11	2005	Naumann et al. [10]	Prospective	FiberKor posts (parallel sided) DentinPost (tapered)	5-6 months (ave: 3.9)	93.3

12	2005	Grandini et al. [23]	Prospective	DT Light posts + direct resin composite	6, 12, 24, and 30 months	No cumulative data given (High performance in general) (100%)

13	2007	Schmitter et al. [8]	Prospective	Glass fiber posts, screw posts	1 year	93.5

14	2007	Cagidiaco et al. [24]	Prospective	DT Light posts	23–25 months	Post debondings: 4.3 Endodontic failures: 3.0

15	2007	Naumann et al. [22]	Prospective	Titanium posts, fiber reinforced posts	2-3 years	100

16	2007	Piovesan et al. [25]	Prospective	Polyethylene fiber posts	97 months	95

17	2008	Preethi and Kala [26]	Prospective	Carbon fiber posts, glass fiber posts, cast posts	1 year	100

18	2008	Mehta and Millar [27]	Prospective	Fibre-White Parapost cemented either by Calibra or Panavia.	38–54 months	Calibra: 64.1 Panavia: 79.5

19	2009	Signore et al. [28]	Retrospective	FibreKor fiber posts (parallel sided or tapered)	5.3 years	98.5

20	2011	Ghavamnasiri et al. [29]	Retrospective	RTD posts (quartz fiber), extensive composite resin	1–6 years	48.8

21	2011	Schmitter et al. [6]	Prospective	ER dentin posts (glass fiber) and titanium screw posts	5 years	71.8

22	2011	Zicari et al. [30]	Prospective	Prefabricated posts Custom-made glass fiber posts	7–37 months	Prefabricated: 91.7 Custom made: 97.2

23	2012	Naumann et al. [31]	Prospective	Glass fiber posts	120 months	63

24	2012	Sterzenbach et al. [5]	Prospective	Glass fiber posts, titanium posts	84 months	Glass fiber posts: 90.2 Titanium posts: 93.5
